# Electronic hand hygiene monitoring systems: Perceptions and behaviors

**DOI:** 10.1017/ash.2022.146

**Published:** 2022-05-16

**Authors:** Rachel Elliott, Jana Shaw, Paul Suits, Emina Fetibegovic, Telisa Stewart, Roger Wong, Julie Briggs

## Abstract

**Background:** Electronic hand hygiene monitoring systems (EHHMSs) are being increasingly utilized to improve hand hygiene outcomes. Following the implementation of an EHHMS at a large, academic medical center, an interdisciplinary team developed a web-based survey to gather information on employee’s perceptions and behaviors surrounding the EHHMS. **Methods:** In total, 1,273 complete responses were collected. Responses were analyzed using Stata version 16 statistical software with 2-tailed tests and .05 significance level. Multivariate logistic regression models were constructed to examine factors associated with negative perceptions of the EHHMS and of wearing the EHHMS radiofrequency identification (RFID) badge. Supporting qualitative analysis was performed using Atlas.ti version 9 software. **Results:** The general sentiment toward the monitoring system was neutral (38%) to negative (37%). The same was true for respondents’ sentiments toward wearing the RFID badge. Of respondents who interact with the system, 48% feel that the system does not capture hand hygiene data accurately. The EHHMS had limited influence on employee’s hand hygiene habits: 27% significant influence and 54% little-to-no influence. Respondents of younger age, those employed as a registered nurse, scientist, physician, or master’s level clinician, and those working at the satellite hospital were significantly more likely to have negative perceptions of the EHHMS. Negative perceptions were also significantly more likely among respondents familiar with the institution’s hand hygiene policy and those who had a negative opinion of seeing the hand hygiene data of others. Negative perceptions of the EHHMS RFID badge were significantly more likely among respondents of younger age, those employed as a registered nurse, scientist, physician, or master’s level clinician, those working at the satellite hospital, and those with a negative perception of seeing the hand hygiene data of others. Employment in a role providing direct patient care and those employed at the institution for >1 year were also significantly more likely to have a negative perception. **Conclusions:** Negative and neutral opinions dominate perceptions of the EHHMS considered in this analysis. Respondents expressed concerns with accuracy of the EHHMS data collection. The system’s limited influence is likely a result of limited familiarity, limited performance feedback, and employee frustration and concerns. These findings provide opportunities for improvement in future implementation of EHHMS. Based on these results, implementation of EHHMS would be best be supported by coordinated backing from administration and leadership, advanced planning and education, and frequent, effective communication. Additional research and evaluation are required to optimize implementation of electronic hand hygiene monitoring systems, with the goal of improving hand hygiene outcomes.

**Funding:** None

**Disclosures:** None

